# Functional Heterogeneity of the Autism Spectrum Disorder‐Associated Gut Microbial Ecosystem Revealed by Fecal Lipopolysaccharides and Bacterial Proteomic Profiling

**DOI:** 10.1002/cbic.70557

**Published:** 2026-09-29

**Authors:** Stefania De Chiara, Angela Di Somma, Valentina Mazziotti, Serena Coppola, Franca Oglio, Lidia Tammaro, Maria Pia Riccio, Carmela Bravaccio, Antonio Molinaro, Roberto Berni Canani, Flaviana Di Lorenzo

**Affiliations:** ^1^ Department of Chemical Sciences University of Naples Federico II Naples Italy; ^2^ CEINGE Advanced Biotechnologies Naples Italy; ^3^ Department of Translational Medical Science University of Naples Federico II Naples Italy; ^4^ National Institute of Biostructure and Biosystems (INBB) Rome Italy

**Keywords:** autism spectrum disorder, bacterial proteins, gut microbiota, lipopolysaccharides, neuroinflammation

## Abstract

The etiology of pediatric autism spectrum disorder (ASD) has been increasingly linked to alterations in the gut‐brain axis, highlighting the intricate bidirectional communication between gut microbiota and central nervous system. The molecular mechanisms of this communication are poorly understood. Here we investigated whether ASD‐associated gut microbiota could exhibit altered inflammatory molecular outputs by integrating chemistry‐driven profiling of fecal lipopolysaccharides (LPS), functional bacterial proteomics, and neuroimmune cellular assays. Structural analyses revealed that LPS from non‐autistic healthy donors (NASD) displayed highly conserved carbohydrate and lipid A signatures dominated by hypo‐acylated *mono*‐phosphorylated species typically associated with immunomodulatory *Bacteroides*‐derived LPS. In contrast, LPS from ASD children exhibited increased structural heterogeneity. These molecular alterations were functionally reflected in human HMC3 microglial cells, where ASD‐derived fecal LPS induced stronger IL‐6 and IL‐8 release compared with NASD‐derived LPS, indicating enhanced neuroinflammatory potential. Functional proteomic profiling disclosed broadly comparable microbial compositions but marked metabolic divergence. These findings suggest that gut microbiota in ASD children are associated with a functionally heterogeneous microbial ecosystem characterized by altered immunostimulatory molecular outputs despite the absence of massive taxonomic shifts, with potential relevance for neuroimmune dysregulation.

## Graphical abstract

Gut microbiota associated with pediatric autism spectrum disorder exhibit functional heterogeneity characterized by altered fecal lipopolysaccharide landscapes, metabolic remodeling, and enhanced microglial inflammatory responses despite largely preserved microbial composition.

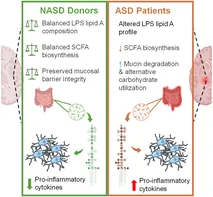

## 1 Introduction

Pediatric autism spectrum disorder (ASD) is a globally prevalent neurodevelopmental condition characterized by persistent difficulties in social communication and interaction, together with restricted interests and repetitive patterns of behavior [1, 2]. Current estimates indicate a rapidly increasing prevalence, with 1%–3.2% of children affected worldwide [1, 2]. Although ASD etiology is highly heterogeneous and involves a complex interplay between genetic susceptibility and environmental influences acting during early development, the molecular mechanisms underlying disease onset and progression remain incompletely understood. Among the pathways increasingly associated with ASD pathophysiology, the gut–brain axis has turned into a key area for study. This bidirectional communication network comprises the gastrointestinal tract, the gut microbiota, the immune system, and the central nervous system, allowing microbial signals to influence neurodevelopment, brain function, and behavior [3]. Consistent with this concept, alterations of gut microbial communities, referred to as dysbiosis, are frequently reported in pediatric patients with ASD [4], suggesting that microbiota‐host interactions may contribute to disease‐associated neuroimmune abnormalities. In particular, these microbiota alterations have been associated with immune dysfunction, elevated levels of pro‐inflammatory cytokines, and microglial activation, all of which have been proposed as contributors to the pathophysiology and severity of core ASD symptoms [5].

Despite growing interest in ASD‐associated dysbiosis, most studies have focused primarily on describing taxonomic differences through elegant and highly informative multi‐omics approaches [6, 7, 8]. While these investigations have identified key microbial signatures associated with ASD, on the other hand, they provide indirect information on the biological activities ultimately experienced by the host. As a result, it remains unclear whether ASD‐associated microbial ecosystems differ not only in composition but also in the nature of the immune‐active molecular signals they deliver. In this scenario, lipopolysaccharides (LPS) are particularly relevant candidates to be investigated. As major components of the outer membrane of Gram‐negative bacteria and primary ligands of the innate immune receptor Toll‐like receptor 4 (TLR4), LPS molecules are one of the most abundant interfaces between the gut microbiota and the host immune system [9]. Importantly, increasing evidence indicates that LPS molecules derived from the gut microbiota display remarkable structural diversity, including within their conserved lipid A moiety, thus resulting in profoundly different immunological activities ranging from potent inflammatory stimulation to immune modulation [9, 10, 11]. Whether ASD‐associated Gram‐negative microbial communities generate structurally and functionally distinct LPS ever, remains largely unexplored.

To address this question, we combined chemistry‐driven profiling of fecal LPS, functional bacterial proteomics, and neuroimmune cellular assays. Here, we provide evidence that ASD‐associated gut microbiota display distinct structural and functional features, characterized by increased heterogeneity of the fecal LPS pool, altered microbial metabolic outputs, and enhanced pro‐inflammatory neuroimmune signaling. Overall, these findings support a broader scenario in which increased functional heterogeneity of the ASD‐associated microbial ecosystem is accompanied by altered inflammatory signaling landscapes with potential relevance for neuroimmune dysregulation and neurodevelopment.

## 2 Results and Discussion

### 2.1 Fecal LPS in NASD and ASD Samples Display Distinct Chemical Signatures

To investigate the structural and functional features of gut microbiota‐derived LPS in ASD, fecal samples from 12 Caucasian children of both sexes (age range 2–7 years), with a confirmed diagnosis of ASD (based on DSM‐5 criteria) and from 12 age‐ and sex‐matched non‐ASD healthy controls (NASD) were analyzed. The stool samples were previously collected in sterile vials from participants of the NAFRA (Nutritional Status and Adverse Food Reactions in children with Autism Spectrum Disorder) project [12] and immediately stored at −80 °C in the Anemone biobank at the Ceinge Advanced Biotechnologies Research Center of the University of Naples Federico II. We excluded subjects with use of pre‐ or pro‐ or postbiotic products and/or antibiotics in the previous 4 weeks; allergic diseases; autoimmune diseases; obesity; immunodeficiencies; chronic inflammatory bowel diseases; celiac disease; cystic fibrosis; metabolic diseases; lactose intolerance; malignancy; and malformations of the gastrointestinal tract. Written informed consent was obtained from the parents/guardians of each subject. Fecal samples were processed to obtain supernatants, which were then subjected to a modified enzyme‐hot phenol/water extraction procedure, followed by multiple purification and repurification steps to remove potential co‐extracted immunostimulatory contaminants, including lipoproteins, lipopeptides, and phospholipids [13]. Purified fecal LPS extracts were then subjected to compositional analyses. Monosaccharide profiling, performed after chemical derivatization and GC–MS analysis, revealed a largely conserved carbohydrate signature across NASD samples (Table S1). In particular, heptose residues were not detected in any NASD extract. This feature is consistent with our previous analyses on a similar cohort [13] and indicative of a predominant contribution of *Bacteroides*‐derived LPS, as LPS from this genus are known to lack heptose units in the inner core region [11, 13, 14, 15, 16]. These profiles were also characterized by the presence of common hexoses and hexosamines together with 3‐deoxy‐D‐*manno*‐oct‐2‐ulosonic acid (Kdo, marker of LPS molecules), forming a recurring “sugar barcode” consistent among most NASD samples. In contrast, ASD‐derived LPS extracts displayed a more heterogeneous monosaccharide composition, suggesting a broader diversity in the structural origin of the LPS molecules (Table S1). Notably, while heptose residues were not detected in NASD extracts, they were observed in a subset of ASD samples (Table S1). This finding preliminarily indicated a reduced predominance of *Bacteroides*‐associated LPS signatures in ASD samples, thereby allowing structurally minor LPS glycoforms containing heptose residues to become detectable.

### 2.2 Fecal Lipid A Profiles From ASD Samples Display Increased Heterogeneity

To obtain a rapid structural and functional overview of the immunostimulatory potential of fecal LPS, we focused on the lipid A portion, the bioactive moiety primarily responsible for TLR4/MD‐2 activation and downstream inflammatory signaling [9]. Lipid A fractions were isolated directly from aliquots of each fecal supernatant using a micro‐scale extraction protocol as previously described [13]. The resulting lipid A mixtures were analyzed by negative‐ion MALDI‐TOF mass spectrometry. Lipid A spectra obtained from NASD samples were highly reproducible across children (Figure 1). All NASD spectra were characterized by ions with *m/z* values below approximately 1720, consistent with the occurrence of hypo‐acylated (i.e., less than six acyl chains) lipid A species, in agreement with previous results [13]. In particular, two main clusters of ions were consistently detected across all NASD samples. These signals were assigned to *mono*‐phosphorylated tetra‐ and penta‐acylated lipid A species, a structural pattern previously associated with lipid A from commensal gut bacteria, particularly members of the genus *Bacteroides* [13, 14, 15, 16]. Within each cluster, mass differences of 14 amu were observed, reflecting variations in acyl chain length and consistent with the known structural heterogeneity of *Bacteroides*‐type lipid A [14, 15, 16]. Overall, the high similarity of the lipid A mass spectral profiles across NASD samples supports the presence of a conserved fecal LPS profile dominated by hypo‐acylated lipid A species, typically associated with low pro‐inflammatory potential. A minor exception was observed for NASD10 and NASD11, which showed an apparent reduction in the clusters attributed to *Bacteroides*‐type lipid A (Figure 1). Consistently, and interestingly, these samples also lacked fucose, a monosaccharide reported in several *Bacteroides* LPS structures described to date [14, 15] (Table S1).

**FIGURE 1:**
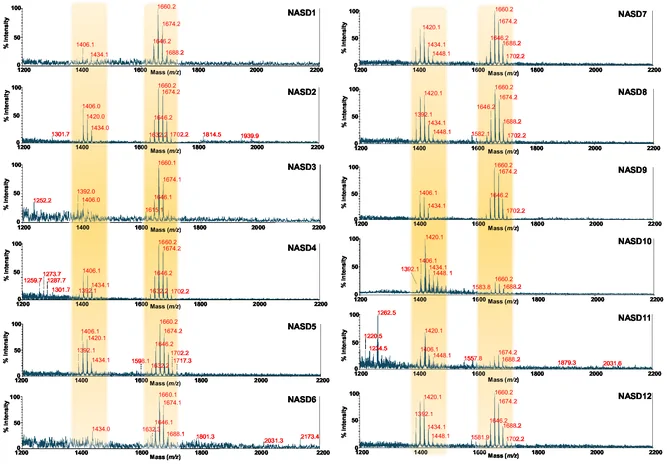
MALDI‐TOF mass spectral profile of lipid A mixture extracted from fecal supernatants of NASD children. MALDI‐TOF MS spectra were recorded in negative ion polarity. Light orange boxes emphasize the clusters of peaks attributed to *mono*‐phosphorylated tetra‐ (around *m/z* 1420) and penta‐acylated (around *m/z* 1660) lipid A species from *Bacteroides* sp. Spectra were labeled “NASD” followed by a number where NASD refers to a healthy control child while the number is unique for each child and is used to trace their information in the collection of samples of the NAFRA project.

By contrast, lipid A spectra obtained from ASD fecal samples displayed a more heterogeneous pattern, although the overall spectral features remained largely comparable to those observed in NASD children (Figure 2). In most cases, the dominant clusters of ions below *m/z* ~1720, corresponding to *mono*‐phosphorylated tetra‐ and penta‐acylated lipid A species, were still clearly detectable, indicating the persistence of *Bacteroides*‐type lipid A. However, several ASD samples exhibited additional spectral features not observed in their NASD counterparts. In a subset of children (ASD1, ASD4, ASD9, and ASD12), a relative decrease in the intensity of peaks assigned to *mono*‐phosphorylated penta‐acylated lipid A species (at about *m/z* 1660) was accompanied by a proportional increase in tetra‐acylated forms (at about *m/z* 1420). In addition, a significant cluster of peaks centered at *m/z* ~1259 was observed in a few ASD spectra and assigned to *bis*‐phosphorylated tri‐acylated lipid A species. Moreover, in several cases (ASD1, ASD3, ASD4, ASD8, ASD9, ASD10, and ASD11), key ions with *m/z* values above 1720 were detected. These higher‐mass ions are indicative of *bis*‐phosphorylated and/or more highly acylated lipid A species, structural features commonly associated with members of the Gammaproteobacteria and with increased pro‐inflammatory potential [11]. Finally, most ASD spectra displayed lower signal‐to‐noise ratios compared to their NASD counterparts. Together with the monosaccharide compositional analyses, this observation supports the presence of a more heterogeneous Gram‐negative molecular scenario in ASD samples, in which multiple lipid A chemotypes with rather comparable relative abundances may reduce the predominance of a single *Bacteroides*‐associated structural signature.

**FIGURE 2:**
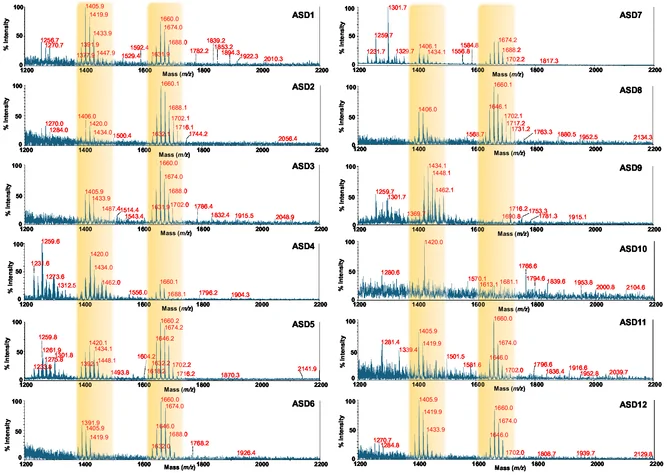
MALDI‐TOF mass spectral profile of lipid A mixture extracted from fecal supernatants of ASD children. MALDI‐TOF MS spectra were recorded in negative ion polarity. Light orange boxes emphasize the clusters of peaks attributed to *mono*‐phosphorylated tetra‐ (around *m/z* 1420) and penta‐acylated (around *m/z* 1660) lipid A species from *Bacteroides* sp. Spectra were labeled “ASD” followed by a number where ASD refers to a child with ASD while the number is unique for each child and is used to trace their information in the collection of samples of the NAFRA project.

### 2.3 Proteomic Analysis Reveals Functional Differences of the ASD‐Associated Microbiota

Identification of bacterial proteins in fecal samples from NASD and ASD children provided complementary insight into both the taxonomic composition and the functional metabolic activities of the gut microbiota. Consistent with the LPS analyses, comparative proteomic profiling revealed broadly similar microbial communities at the taxonomic level between ASD and NASD samples (Figure 3), supporting the absence of major compositional rearrangements within the bacterial ecosystem. However, despite this overall taxonomic similarity, functional profiling revealed marked differences in the distribution of bacterial proteins between the two groups. Identified proteins were classified according to their primary annotated biological function (Table S2), revealing distinct functional signatures associated with the ASD and NASD microbiota.

**FIGURE 3:**
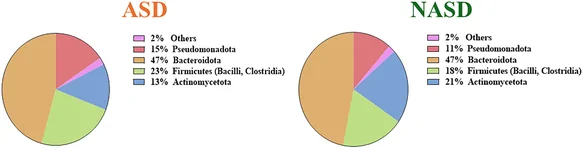
Taxonomic distribution of bacterial proteins identified in fecal samples from ASD and NASD children. Relative abundance of bacteria assigned to the major gut‐associated phyla identified by LC–MS/MS‐based proteomic analysis. For each cohort, percentages were calculated by summing the number of proteins assigned to each phylum across individual samples and expressing this value relative to the total number of bacterial proteins assigned across all phyla within that cohort.

As shown in Figure 4, ASD fecal samples were enriched in enzymes involved in carbohydrate metabolism and glycolysis, including multiple isomerases and dehydrogenases, together with stress‐response proteins, such as superoxide dismutases and catalases. Many of these proteins originated from taxa commonly associated with opportunistic or pro‐inflammatory microbial states, including members of the Firmicutes phylum. In contrast, NASD samples displayed a higher representation of proteins involved in fatty acid metabolism, particularly butyrate and acetate biosynthesis, as well as amino acid biosynthesis pathways, many of which were derived from commensal bacteria linked to intestinal homeostasis. These findings suggest that ASD‐associated microbiota alterations may be more strongly reflected at the functional and metabolic level than through major taxonomic shifts, supporting a model in which changes in microbiota‐derived molecular activities contribute to altered inflammatory and neuroimmune signaling.

**FIGURE 4:**
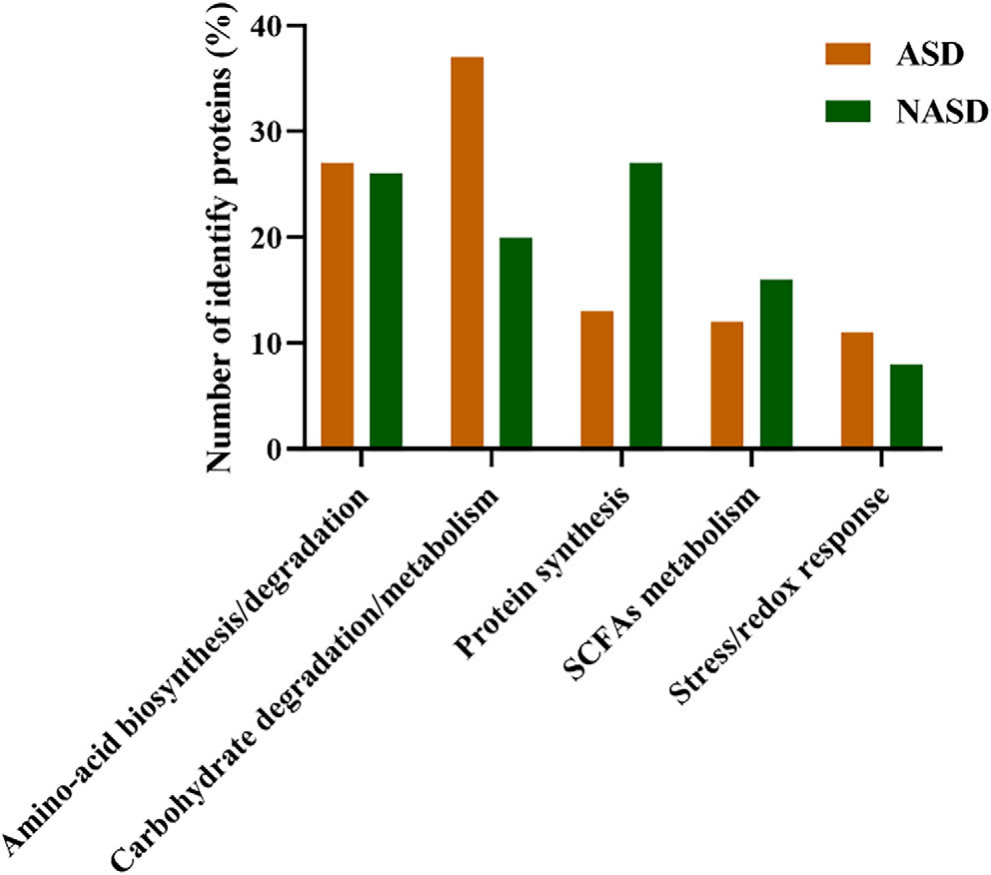
Functional classification of fecal bacterial proteins in ASD and NASD children. Bar plot showing the distribution of bacterial proteins and grouped into major functional categories based on their primary annotated function. Protein classification was based on presence across samples.

### 2.4 Differential Protein Expression Analysis Highlights Metabolic Imbalance in ASD‐Associated Microbiota

The functional differences exhibited by the bacterial proteins prompted us to perform a quantitative analysis of the proteomic data using the label‐free procedure [17] to highlight differential protein expression in ASD and NASD fecal samples. Approximately 1000 bacterial proteins were identified and quantitatively evaluated following statistical analysis using Perseus software (Table S3). A distinct subset of significantly modulated proteins confirmed a marked functional divergence between ASD and NASD groups despite the largely comparable taxonomic composition observed in the two groups. The up‐ and downregulated proteins are shown in a volcano plot (Figure 5). A detailed investigation of the down‐regulated proteins in ASD samples indicated a significant reduction in key enzymes involved in the biosynthesis of SCFAs, particularly butyrate and acetate. Notably, 3‐hydroxybutyryl‐CoA dehydrogenase (hbd), a central enzyme of the acetyl‐CoA pathway for butyrate production, was significantly decreased compared to that in NASD samples. Given the established role of butyrate in maintaining intestinal epithelial integrity and modulating neuroimmune signaling through histone deacetylase (HDAC) inhibition, this reduction suggests a diminished neuroprotective and anti‐inflammatory potential of the ASD‐associated microbiota [18, 19]. Consistently, acetate kinase (ackA) and phosphoketolase (xfp), enzymes involved in acetate production and characteristics of bifidobacterial metabolism, were also significantly downregulated. This pattern suggests impaired acetate‐mediated cross‐feeding interactions that normally sustain butyrate‐producing commensal species, further compromising SCFA homeostasis. When the up‐regulated proteins were examined, a significant increase in proteins associated with mucin degradation and alternative carbohydrate utilization pathways was detected in ASD samples. In particular, sialidase (nanH) and L‐fucose isomerase (fucI) were markedly increased. NanH catalyzes the cleavage of terminal sialic acid residues from mucosal glycoconjugates, suggesting enhanced erosion of the protective mucus layer and potential weakening of gut barrier integrity [20, 21]. Similarly, increased fucI abundance indicates the activation of the fucose utilization pathway, which can drive propionate production. Although propionate exerts regulatory functions at physiological concentrations, its excessive production has been associated with neuroinflammatory responses and ASD‐like phenotypes in experimental models [22].

**FIGURE 5:**
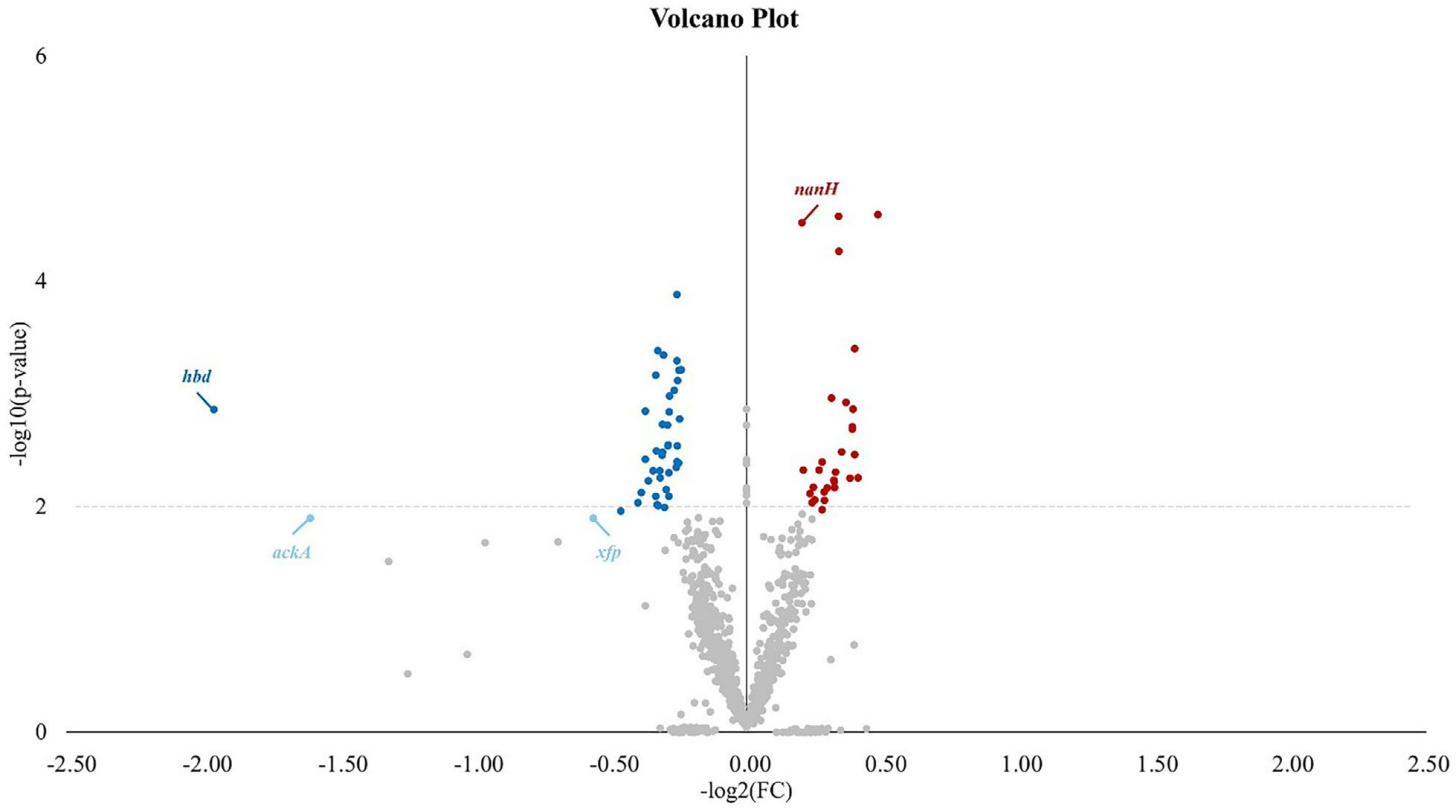
Volcano plot representation of differentially expressed proteins identified by label‐free proteomics (ASD/NASD). The *x*‐axis shows log2 fold change, and the *y*‐axis represents –log10 (*p*‐value). Proteins significantly up‐regulated in ASD samples are shown in red, whereas significantly down‐regulated proteins are shown in blue.

### 2.5 Fecal LPS From ASD Children Trigger a Robust Pro‐Inflammatory Cytokine Response

Microglia dysregulation has been implicated in the initiation and maintenance of pro‐inflammatory processes that may contribute to ASD pathogenesis and progression [23, 24]. To investigate the neuroinflammatory potential of fecal LPS, we employed the human microglial HMC3 cell line, a widely used in vitro model for studying neuroimmune responses [25]. The morphology of HMC3 cells was first evaluated by brightfield phase‐contrast microscopy (Figure 6A), after which cells were exposed for 24 h to increasing concentrations (0.1–100 ng/mL) of two pooled fecal LPS preparations obtained from the ASD and NASD cohorts. Cell viability, assessed by the MTT assay, was not substantially affected by LPS treatment under most conditions compared with non‐stimulated (NS) cells (Figure 6B). However, at the highest concentration tested (100 ng/mL), ASD‐derived LPS induced a significant reduction in cell viability relative to NASD‐derived LPS (*p* = 0.0001; Figure 6B). To further evaluate the inflammatory potential of gut microbiota‐derived LPS, pro‐inflammatory IL‐8 and IL‐6 release by HMC3 cells was quantified by an ELISA. ASD‐derived LPS induced a robust and concentration‐dependent increase in both cytokines across all tested concentrations, whereas NASD‐derived LPS elicited only modest responses, reaching statistical significance predominantly at the highest concentration tested (Figure 6C,D). Importantly, cytokine production was consistently higher in cells stimulated with ASD‐derived LPS compared with NASD‐derived LPS at the corresponding concentrations, indicating an enhanced pro‐inflammatory potential of the ASD‐associated LPS pool.

**FIGURE 6:**
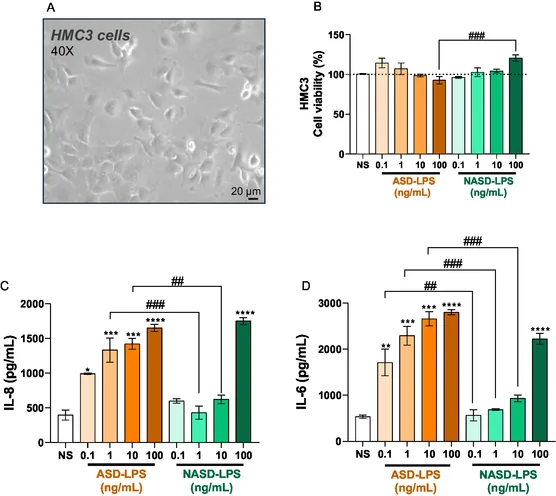
Effects of fecal LPS on HMC3 microglia cell viability and activation. (A) Bright‐field image of HMC3 cells (40× magnification, scale bar: 20 µm). (B) HMC3 microglia cell viability after 24 h exposure to increasing concentrations (0.1, 1, 10, 100 ng/mL) of the pooled fecal LPS preparations obtained from the ASD and NASD cohorts. Cell viability was expressed as % relative to non‐stimulated (NS) cells and assessed by MTT assay (570 nm). NS cells were also considered as negative control. (C,D) Quantification of (C) IL‐8 and (D) IL‐6 levels following LPSs treatment, measured by DuoSet‐ELISA kit. Three independent experiments (*n* = 3) were performed, with each experimental condition tested in triplicate wells within each experiment. Statistical significance was assessed by ordinary one‐way ANOVA followed by Tukey's multiple‐comparison test. *p < 0.05, **p < 0.01, ***p < 0.001, ****p < 0.0001 versus NS; ^#^ *p* < 0.05, ^###^ *p* < 0.001, ^####^ *p* < 0.0001, versus LPS.

### 2.6 Neuronal Responses to ASD‐ and NASD‐Associated Fecal LPS

ASD has been associated with neuronal abnormalities, including altered cell proliferation and differentiation, impaired synaptic development, and reduced neuronal activity [26]. Microglia play a key role in maintaining CNS homeostasis by supporting neurogenesis and regulating synaptic pruning, whereas their dysregulation may contribute to neuronal impairment through the release of pro‐inflammatory cytokines and cytotoxic mediators [27, 28]. To investigate whether microglial activation induced by fecal LPS could indirectly affect neuronal cells, differentiated SH‐SY5Y cells were exposed to conditioned medium (CM) derived from HMC3 microglia previously stimulated with LPS isolated from fecal samples. SH‐SY5Y differentiation was achieved using retinoic acid (RA) and brain‐derived neurotrophic factor (BDNF), as previously described [29, 30], and neuronal morphology was confirmed again by bright‐field phase‐contrast microscopy (Figure 7A). Following 24 h exposure to CM generated from HMC3 cells stimulated with increasing concentrations of LPS (0.1–100 ng/mL), SH‐SY5Y viability was assessed by the MTT assay. CM treatment did not significantly affect neuronal viability under any condition, although CM derived from ASD‐associated LPS showed a tendency toward reduced viability compared with NASD‐derived CM (Figure 7B). Based on the strong cytokine release profile observed in HMC3 cells, 100 ng/mL was selected for subsequent neuronal experiments, as it provides a robust inflammatory environment through CM while still preserving cell viability, thus allowing the assessment of potential differences in neuronal responses to CM generated following stimulation with ASD‐ and NASD‐derived LPS. To this aim, the expression of microtubule‐associated protein 2 (MAP2), a marker associated with neuronal differentiation and dendritic integrity, was evaluated by immunofluorescence and Western blot analysis. Differentiated SH‐SY5Y cells exposed to CM derived from NASD‐associated LPS showed a tendency toward higher MAP2+ cells density compared with those treated with CM generated from ASD‐derived LPS (Figure 7C). Quantification of the MAP2 immunofluorescence signal showed a similar trend (Figure 7D), which was also paralleled by higher MAP2 protein levels detected by Western blot analysis in cells exposed to NASD‐derived CM (Figure 7E). Although these differences did not reach statistical significance, the concordant direction of the changes observed across cell viability, MAP2 immunofluorescence, and MAP2 protein expression suggests a potential differential influence of the microglial responses elicited by ASD‐ and NASD‐associated fecal LPS on the neuronal environment. These observations should nevertheless be interpreted as trends and warrant further investigation.

**FIGURE 7:**
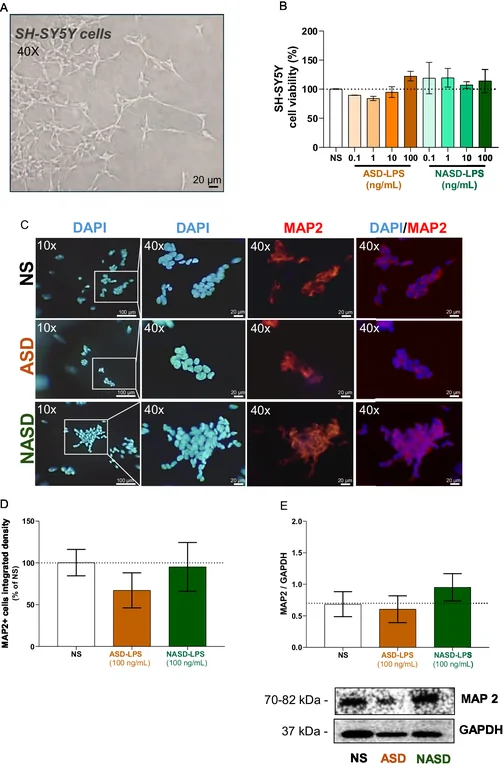
Effects of conditioned media (CM) from fecal LPS‐treated HMC3 microglial on SH‐SY5Y viability and differentiation. (A) Bright‐field image of differentiated SH‐SY5Y cells (40× magnification, scale bar: 20 µm). (B) Cell viability of SH‐SY5Y neurons after 24 h exposure to CM from HMC3 microglia previously stimulated with 0.1, 1, 10, 100 ng/mL of the pooled fecal LPS preparations obtained from the ASD and NASD cohorts. Cell viability was expressed as % relative to non‐stimulated (NS) cells and assessed by MTT assay (570 nm). (C) Representative integrated immunofluorescence images combining MAP2 (red) and DAPI (blue) channels, showing MAP2 immunoreactivity in SH‐SY5Y cells exposed to CM from HMC3 cells treated with 100 ng/mL fecal. The integration of MAP2 and DAPI channels provides both neuronal morphology and nuclear localization within the same composite image. Original magnification: 10× (scale bar, 100 µm); zoomed‐in image: 40× (scale bar, 20 µm). (D) Quantitative MAP2 immunofluorescence analysis performed using ImageJ software (version 1.53t), MAP2 cells integrated density was expressed as % relative to NS. (E) Western blot analysis of MAP2 protein levels in SH‐SY5Y cells exposed to CM from HMC3 cells treated with 100 ng/mL of fecal LPS. Data were presented as mean ± SEM and NS cells served as negative control. Three independent experiments (*n* = 3) were performed, with each experimental condition tested in triplicate wells within each experiment. Statistical significance was assessed by ordinary one‐way ANOVA followed by Tukey's multiple‐comparison test.

## 3 Conclusion

Growing evidence supports the involvement of gut dysbiosis and alterations in the gut‐brain axis in the onset and progression of ASD [31]. Although several studies have focused on variations in gut microbiota composition and metabolite profiles in ASD, the impact of these alterations between ASD and NASD groups remains incompletely understood, and causal mechanisms are still largely unresolved. In this study, we investigated the gut microbiota of ASD and NASD children at the molecular level through two complementary approaches, integrating structural analysis of LPS with functional profiling. This strategy enabled not only the identification of bacterial components present in the samples but also the definition of their functional potential.

The structural characterization of gut microbiota‐derived LPS from neurotypical healthy children revealed a highly conserved carbohydrate and lipid A signature. The absence of detectable heptose residues and the presence of a recurring monosaccharide “barcode” are consistent with a predominant contribution of *Bacteroides*‐derived LPS. This observation was further supported by lipid A mass spectral profiles, which were highly reproducible across NASD children and dominated by *mono*‐phosphorylated tetra‐ and penta‐acylated species, a structural pattern typically associated with commensal gut bacteria and low pro‐inflammatory potential [14, 15, 16]. By contrast, although most ASD lipid A spectra retained features typical of NASD‐associated profiles, the intermittent detection of higher‐mass, more highly acylated/phosphorylated species, together with an increased contribution of tetra‐acylated and tri‐acylated forms with reduced or negligible TLR4 agonistic activity, indicates a disruption of the balanced LPS lipid A landscape observed in the NASD condition. In this context, the predominance of *mono*‐phosphorylated penta‐acylated lipid A species is characteristic of *Bacteroides* sp. and associated with immunomodulatory properties; it appears to be partially altered, suggesting that ASD‐associated LPS profiles are not shifted toward a more pro‐inflammatory state but rather reflect a loss of structural equilibrium within the lipid A pool. This broader structural heterogeneity in fact may reflect the ecological instability increasingly described in the ASD‐associated gut microbiota [32]. Heightened sensitivity to dietary, environmental, or inflammatory perturbations could promote fluctuating contributions of Gram‐negative taxa expressing distinct lipid A chemotypes. However, our data directly demonstrate greater interindividual heterogeneity rather than temporal fluctuations within individual subjects. In this frame, ASD may therefore be associated with a more heterogeneous microbial ecosystem in which inter‐individual variation in microbiota‐derived immune‐active molecules may increase the likelihood of dysregulated inflammatory responses rather than reflecting a persistent and uniformly pro‐inflammatory microbiota. Strikingly, and consistent with this hypothesis, the functional relevance of these structural differences was evident in microglial assays. Fecal LPS derived from ASD samples induced a significantly stronger release of IL‐6 and IL‐8 in HMC3 cells compared with LPS from healthy children despite the overall similarity in lipid A profiles between groups. This observation suggests that even relatively minor variations in the relative abundance of specific lipid A species may be sufficient to alter the immunological output of the LPS mixture. Consistently, conditioned media from HMC3 microglia stimulated with ASD‐derived fecal LPS showed non‐significant trends toward reduced cell viability and MAP2 expression in differentiated SH‐SY5Y cells compared with NASD‐derived LPS. Although these observations do not demonstrate a direct effect on neuronal cells, their concordant direction across different readouts raises the possibility that the distinct microglial responses elicited by ASD‐associated LPS may translate into a less‐supportive neuronal environment, a hypothesis that warrants further investigation.

In addition, proteomic profiling revealed marked differences between the two groups. ASD samples were enriched in proteins associated with carbohydrate metabolism, glycolysis, and stress‐response pathways, whereas NASD samples showed a higher representation of proteins involved in fatty acid metabolism and amino acid biosynthesis. Quantitative proteomic analysis confirmed this divergence, highlighting a reduction in key enzymes involved in SCFA biosynthesis in ASD samples, together with an increased abundance of proteins associated with mucin degradation and alternative carbohydrate utilization [33]. This functional shift suggests a lower representation of SCFA‐producing, homeostasis‐associated microbial activities and a greater representation of pathways linked to mucosal glycan utilization and potentially pro‐inflammatory metabolic outputs [34]. In contrast, NASD samples were enriched in enzymes associated with butyrate and acetate biosynthesis, reinforcing the presence of a metabolically balanced and homeostasis‐supporting microbiota. Interestingly, this metabolic divergence paralleled the increased heterogeneity observed within the lipid A pool. Although major taxonomic rearrangements were not detected, both proteomic and LPS analyses support a model in which ASD‐associated microbiota exhibit greater functional heterogeneity, characterized by increased inter‐individual variability in microbiota‐derived immune‐active molecules together with a diminished predominance of homeostasis‐associated activities.

Overall, our findings indicate that ASD‐associated gut microbiota alterations may be better reflected by changes in microbial molecular outputs than by profound compositional shifts. In this context, microbiota‐derived LPS represents a relevant interface linking microbial ecology and host immune activation. By demonstrating that relatively subtle variations in lipid A structural features are associated with measurable differences in microglial activation, together with concordant trends in downstream neuronal responses, this study highlights the importance of considering the qualitative composition of the LPS pool rather than its mere presence. More broadly, these findings also raise the possibility that the structural features of microbiota‐derived LPS may represent informative molecular biomarkers of microbiota function and, in the longer term, potential targets for future microbiota‐directed interventions.

Limitations should be considered when interpreting the present findings. First, all molecular analyses were performed on fecal material, which represents a proxy of the intestinal microbial ecosystem and may not fully capture the microbiota and microbial products directly associated with the intestinal mucosa. Consequently, the LPS and proteomic signatures described here may only partially represent the immune‐active molecules present at the host‐microbiota interface. Future studies integrating mucosal sampling will be necessary to determine whether the functional heterogeneity observed in fecal microbial outputs is also reflected on the mucosal surface. Nevertheless, the findings of this study support the idea that integrating chemical and functional characterization of microbiota‐derived molecules may provide additional insight into the relationship between gut microbiota and neurodevelopment in ASD.

## 4 Experimental Section

### 4.1 Extraction and Purification of Fecal LPS

The fecal samples analyzed in this study were obtained from children enrolled in the NAFRA (Nutritional Status and Adverse Food Reactions in children with Autism Spectrum Disorder) project [12]. The study was approved by the Ethics Committee of the University of Naples, Federico II, and was conducted in accordance with the Declaration of Helsinki (Fortaleza revision, 2013), the principles of Good Clinical Practice (CPMP/ICH/135/95), and the applicable European and Italian regulations on data protection. Written informed consent was obtained from the parents or legal guardians of all participating children prior to enrolment and sample collection. The NAFRA project was registered at ClinicalTrials.gov (NCT04719923). For each subject, 200 mg of lyophilized fecal supernatant was subjected to sequential enzymatic treatments prior to LPS extraction. Samples were incubated with DNase and RNase (Roth) at 37 °C for 16 h under constant stirring, followed by protease digestion at 56 °C for 16 h under constant stirring [13]. Digested preparations were dialyzed against distilled water to remove residual proteins and nucleic acids and then collected and freeze‐dried. LPS was extracted using the hot phenol/water method [35]. Aqueous and phenolic phases were screened by SDS‐PAGE, followed by silver staining. LPS was consistently detected in the aqueous phases, which were therefore retained for further processing. The aqueous fractions were ultracentrifuged (4 °C, 208,000×*g*, 24 h), and the resulting pellets were collected and further purified by size‐exclusion chromatography on Sephacryl S‐300 (GE Healthcare; 1.5 × 20 cm) using 50 mM NH_4_HCO_3_ as the eluent. To remove co‐extracted phospholipids, LPS preparations were washed five times with CHCl_3_/CH_3_OH (1:2, v/v) and CHCl_3_/CH_3_OH/H_2_O (3:2:0.25, v/v/v) [36]. After solvent removal and repeated lyophilization cycles, samples underwent an additional re‐purification step according to Hirschfeld et al. [37] to eliminate traces of lipoproteins/lipopeptides. Purity was assessed by SDS‐PAGE using both silver nitrate and Coomassie Brilliant Blue staining.

### 4.2 Compositional Analyses of Fecal LPS

Monosaccharide composition was determined by GC–MS analysis of acetylated *O*‐methyl glycoside (AMG) derivatives [38]. Briefly, purified fecal LPS was methanolyzed with HCl/CH3OH (1.25 M, 85 °C, 16 h), followed by acetylation with acetic anhydride in pyridine (85 °C, 25 min). Sugar absolute configuration was assigned through preparation and GC–MS analysis of acetylated *O*‐octyl glycoside (OGA) derivatives and comparison with authentic standards, as previously described [39]. All derivatives were analyzed using an Agilent 7820A GC system coupled to a 5977B mass‐selective detector equipped with an HP‐5 capillary column (30 m × 0.25 mm i.d.; He, 1 mL/min).

### 4.3 MALDI‐TOF MS Analysis of Fecal Lipid A

Lipid A was isolated directly from fecal material starting from approximately 1 mg of dried fecal supernatant. Samples were sequentially washed with CHCl_3_/CH_3_OH (1:2, v/v) and CHCl_3_/CH_3_OH/H_2_O (3:2:0.25, v/v/v), with centrifugation steps (1200×*g*, 30 min) between washes. Each wash fraction was dried, weighed, and inspected by MALDI‐TOF MS prior to disposal. The washed material was then processed as previously described [13]. MALDI‐TOF MS analyses were performed in the reflectron mode in both negative‐ and positive‐ion polarity using an AB SCIEX TOF/TOF 5800 instrument (Nd:YAG laser, 349 nm) equipped with delayed extraction. 2,4,6‐trihydroxyacetophenone (THAP) was used as the matrix prepared in CH_3_OH/0.1% TFA/acetonitrile (7:2:1, v/v/v) [40]. Alternative matrices (DHB, α‐cyano‐4‐hydroxycinnamic acid, and 5‐chloro‐2‐mercaptobenzothiazole) were evaluated but resulted in the loss of signals for several samples. After spotting, the mixtures were air‐dried at room temperature prior to acquisition. Lipid A obtained by mild acid hydrolysis of *E. coli* O127:B8 LPS (Sigma–Aldrich) was used as a reference standard. For MS acquisition, each spectrum represented the sum of 3000 laser shots; MS/MS spectra were acquired by summing 5000–7000 shots. Multiple positions per spot were sampled across the *m*/*z* 1200–2200 range. Data were calibrated and processed using Data Explorer software. All measurements were performed in technical triplicate and repeated on three independent days using newly extracted fecal lipid A preparations.

### 4.4 Proteomic Sample Preparation

Fecal pellets were suspended in 1× PBS and centrifuged at 2000 rpm for 5 min to remove the insoluble material. The resulting supernatants were subsequently centrifuged at high speed (20,000 rpm, 30 min, 4 °C) to pellet bacterial cells. Pellets were resuspended in lysis buffer containing 50 mM NH_4_HCO_3_ and 5% SDS, followed by centrifugation at 16,000 rpm for 10 min to collect clarified protein extracts. Proteins were isolated by CHCl_3_/CH_3_OH extraction, as previously described [41]. Protein concentration was determined using the BCA Protein Assay Kit (Thermo Scientific, #23227), and 50 μg of total protein was subjected to tryptic digestion using S‐Trap Micro Spin Columns according to the manufacturer's instructions (Protifi, Huntington, NY, USA) [17].

### 4.5 Proteomic Analysis by Mass Spectrometry

Hydrolyzed peptide mixtures were analyzed on an Orbitrap Fusion Tribrid mass spectrometer (Thermo Fisher Scientific, Waltham, Massachusetts, USA). Peptides were desalted on a trap column (Acclaim PepMap 100 C18, Thermo Fisher Scientific) and then separated on a 20‐cm‐long silica capillary (Silica Tips FS 360‐75‐8, New Objective, MA, United States) packed in‐house with a C18, 5 μm, 100 Å resin (Michrom BioResources, CA, United States). The analytical separation was run for 91 min using a gradient of buffer A (95% water, 5% acetonitrile, and 0.1% formic acid) and buffer B (95% acetonitrile, 5% water, and 0.1% formic acid). The gradient was run as follows: buffer B was fixed at 5% for the first 5 min, then was linearly increased to 30% in 60 min and subsequently to 80% in 10 min, then remained at 80% for 5 min, and finally decreased at 5% for a 10‐min long column re‐equilibration step. Data‐dependent acquisition was performed in top‐speed mode (3 s long maximum total cycle): the most intense precursors with charge >1 were selected through a monoisotopic precursor selection (MIPS) filter, quadrupole‐isolated, and fragmented by 30% higher‐energy collision dissociation (HCD). Fragment ions were analyzed in an ion trap with a rapid scan rate. The LC‐MS/MS data were run in triplicate.

Protein identification and quantification based on LC‐MS/MS data were performed using MaxQuant (v 2.4.10) with a reviewed version of UniProtKB/Swiss‐Prot Bacteria containing 264459 sequences as a database for Andromeda search. To focus the analysis on the gut microbiota, proteins assigned to bacterial taxa not typically associated with the human intestinal environment were excluded from downstream interpretation, thereby minimizing potential contributions from environmental contaminants or transient microbial species. Parameters used for protein identification were the following: (i) a minimum of 3 peptides, including at least 1 unique; (ii) allowed variable modifications were methionine oxidation and pyroglutamate formation on N‐terminal glutamine; (iii) accuracy for the first search was set to 10 ppm; and (iv) 0.01 FDR. Statistical analyses were carried out using Perseus software (v 1.6.15.0) to filter out protein contaminants (i.e., reverse and potential contaminants) and those entries with more than 70% of invalid values in at least one group in the LFQ intensity column. The remaining zero values were imputed according to the Gaussian method [42]. A Student's t‐test was performed with a 1% Benjamini–Hochberg‐corrected *p*‐value cutoff. The fold changes (FC) were calculated as the mean difference of the protein log2 LFQ intensities in ASD versus NASD (ASD/NASD). The differentially expressed proteins were considered those with log2FC<−0.5 and log2FC >0.5 as down‐ and up‐regulated, respectively.

### 4.6 Cell Cultures

Human embryonic microglial cells (HMC3, #300102) and human neuroblastoma cells (SH‐SY5Y, #300154) were purchased from Cytion Biosciences (Heidelberg, Germany). HMC3 cells were cultured in Dulbecco's modified Eagle's medium (DMEM; Cytion Biosciences, #820400a) containing 3.1 g/L glucose, 1.6 mM L‐glutamine, 15 mM HEPES, 1 mM sodium pyruvate, and 1.2 g/L NaHCO_3_, supplemented with 10% heat‐inactivated fetal bovine serum (FBS HI; Gibco, #10270‐106). SH‐SY5Y cells were maintained in DMEM/F12 (Sigma–Aldrich, Rahway, NJ, United States, #D8437) with 10% FBS HI and 0.1% penicillin/streptomycin (Pen/Strep; Gibco, Grand Island, NY, United States, #15140‐122). Cells were grown at 37 °C in a humidified atmosphere containing 5% CO_2_. For neuronal differentiation, SH‐SY5Y cells were cultured in a medium containing 1% FBS (reduced from 10%) and supplemented daily with 10 μM retinoic acid (RA; Thermo Fisher, #044540.02) and 50 ng/mL brain‐derived neurotrophic factor (BDNF; Cytion Biosciences, #359Z03208) for 7 days in vitro.

### 4.7 Cell Viability Assay in HMC3 Microglia and SH‐SY5Y Neurons

LPS preparations obtained from the subjects within each cohort were combined to generate two pooled LPS preparations, which were used to stimulate cells. HMC3 and SH‐SY5Y cell viability was assessed using the 3‐(4,5‐dimethylthiazol‐2‐yl)‐2,5‐diphenyltetrazolium bromide (MTT) assay. Cells were seeded in 96‐well plates at 1 × 10^4^ cells/well, and after 24 h, HMC3 were treated for an additional 24 h with LPS isolated from fecal samples. SH‐SY5Y cells were instead exposed to conditioned media derived from LPS‐treated HMC3 microglia. After treatment, the medium was removed, and the cells were incubated with MTT (0.25 mg/mL) for 3 h at 37 °C. Formazan crystals in the cells were dissolved with dimethyl sulfoxide (DMSO; Sigma–Aldrich, # 34943), and absorbance was measured at 570 nm using a TECAN Infinite M Plex spectrophotometer (Scientific Laboratory Supplies, UK, #MIC9268).

### 4.8 Cytokines Quantification in HMC3 Supernatants

HMC3 were seeded in 12‐well plates at 1 × 10^5^ cells/well and treated for 24 h with different concentrations of fecal LPS (0.1–100 ng/mL) for 24 h [43]. Supernatants were collected and frozen (−20 °C) for subsequent detection and quantification of cytokines via the ELISA assay. Cytokine levels (pg/mL) in HMC3 supernatants were measured using DuoSet ELISA kits (R&D Systems, Minneapolis, MN, USA) for human IL‐8 (#DY208) and IL‐6 detection (#DY206). The absorbance was measured at 450 nm using a TECAN Infinite M Plex spectrophotometer.

### 4.9 Immunofluorescence

SH‐SY5Y were seeded on glass coverslips placed in a 24‐well plate at a density of 3 × 10^4^ cells/well, differentiated for 7 days, and subsequently exposed for 24 h to CM derived from LPS‐treated HMC3 microglia. After treatment, cells were washed once with 1× PBS and fixed in 4% paraformaldehyde (PFA, Thermo Fisher, # J61899) for 15 min at room temperature (RT). Fixed cells were washed three times with 1× PBS and permeabilized with 1× PBS containing 0.1% Triton X‐100 (PBS‐T) for 10 min at RT. Cells were then saturated with a blocking solution (1% FBS + PBS‐T) for 45 min at RT and then incubated with the rabbit anti‐MAP2 primary antibody (Cell Signaling Technology, #8707, 1:300 in 0.5% FBS + PBST) for 1 h. After three washes with 1× PBS, cells were incubated with AlexaFluor 555‐conjugated goat anti‐rabbit IgG secondary antibody (Life Technologies, Carlsbad, CA, United States, #A‐21429 1:500) for 1 h. Nuclear staining was performed by incubating cells with DAPI (Thermo Fisher Scientific, #62248, 1:1000) for 5 min. Finally, coverslips were washed three times with 1× PBS and mounted using SlowFade Gold Antifade reagent (Thermo Fisher Scientific, #S36937) for microscopic examination. Fluorescence images were acquired using OL3000TFL biological microscope (Zetalab, Italy). For the quantification of MAP2^+^ SH‐SY5Y cell count values, values were expressed as a percentage relative to the NS condition. Immunofluorescence images were analyzed using ImageJ software (version 1.53t, National Institutes of Health, Bethesda, MD, United States).

### 4.10 Western Blot

Cells were seeded and treated for Western blot analysis following the same experimental conditions used for immunofluorescence. After treatment, cells were lysed using RIPA buffer (50 mM Tris–HCl pH 7.4, 150 mM NaCl, 1% NP 40, 0.5% sodium deoxycholate, 0.1% SDS, 2 mM EDTA, and 100X protease inhibitors; Sigma–Aldrich). Lysates were incubated on ice for 20 min, centrifuged at 14 000 rpm for 15 min at 4 °C, and total protein in the supernatants was quantified using the PierceTM BCA protein assay kit (Thermo Scientific, #23227). Equal amounts of protein were electrophoretically separated using 7.5% polyacrylamide gels and transferred to a polyvinylidene fluoride (PVDF) membrane (Bio Rad, Hercules, CA, United States, #1620177). Membranes were blocked with 5% non‐fat dry milk and then incubated overnight at 4  °C with a rabbit anti‐MAP2 primary antibody (Cell Signaling Technology, Danvers, MA, United States, #8707; 1:1000). Mouse anti‐GAPDH (ThermoFisher Scientific, #MA5‐15738, 1:3000) was used as a housekeeping protein to normalize total protein levels. Subsequently, after washing with TBS‐Tween 0.1% (10 min/time), membranes were incubated for 1 h at room temperature with horseradish peroxidase (HRP)‐conjugated secondary antibodies (Merck/Sigma Aldrich, Rahway, NJ, United States). The immunoreactive bands were visualized by an enhanced chemiluminescence (ECL) substrate (GE Healthcare AmershamTM, Amersham, United Kingdom, RPN2106) using the Azure 600 Imaging System (Azure Biosystems, Inc., United States).

### 4.11 Statistical Analysis

All data are expressed as the mean ± SEM and represent at least three independent experiments. Statistical significance comparisons between LPS treatments were performed using one‐way ANOVA, followed by multiple comparison tests. Post hoc analyses were performed with Tukey's multiple comparison test. Analyses were conducted using Prism software version 10.1.1(270) (GraphPad, San Diego, CA, USA), and statistical significance was determined at *p* < 0.05.

## Funding

The study was supported by the Department of Translational Medical Science and the Department of Chemical Sciences of the University of Naples Federico II (Naples, Italy), which received funding from the National Recovery and Resilience Plan (NRRP), Mission 4 Component 2 Investment 1.3—Call for tender No. 341 of 15 March 2022 of Italian Ministry of University and Research funded by the European Union—NextGenerationEU. Project code PE00000003, Concession Decree No. 1550 of 11 October 2022, adopted by the Italian Ministry of University and Research, CUP E63C22002030007, Project title “ON Foods—Research and innovation network on food and nutrition Sustainability, Safety and Security—Working ON Foods” and from the Italian Ministry of Health–Health Operational Plan Trajectory 5‐Line of action “Creation of an action program for the fight against malnutrition in all its forms and for the dissemination of the principles of the Mediterranean diet” (Mediterranean Diet for Human Health Lab, “MeDiHealthLab”, code T5‐AN‐07, CUP E63C22002570006). The study was also financially supported by European Research Council (ERC) under the Horizon Europe program under Grant Agreement No, 101039841 (DEBUGGING LPS) to F.D.L. and Grant Agreement No. 101198490 (LPS‐CODE) to A.M. Financial support also came from Ministry of Education, Universities and Research, PRIN MUR 2022 (2022SHW3KY) and PRIN MUR PNRR 2022 (P202293ZMC) to F.D.L.

## Conflicts of Interest

The authors declare no conflicts of interest.

## Supporting information

Supplementary Material

## Data Availability

The data that support the findings of this study are available in the Supporting Information of this article.
