# Supplementary material for: Functional Heterogeneity of the Autism Spectrum Disorder‐Associated Gut Microbial Ecosystem Revealed by Fecal Lipopolysaccharides and Bacterial Proteomic Profiling
Source: Chembiochem. 2026 Sep 29;27(19):e70557. doi: 10.1002/cbic.70557 (PMC13623111; doi:10.1002/cbic.70557)
Supplement: Supplementary file 1 [file CBIC-27-e70557-s001.pdf]

Supporting Information

Functional heterogeneity of the autism spectrum disorder (ASD)-associated gut microbial ecosystem revealed by fecal lipopolysaccharides and bacterial proteomic profiling

Stefania De Chiara, Angela Di Somma, Valentina Mazziotti, Serena Coppola, Franca Oglio, Lidia Tammaro, Maria Pia Riccio, Carmela Bravaccio, Antonio Molinaro, Roberto Berni Canani, Flaviana Di Lorenzo

Table S1. Monosaccharide composition analysis of NASD and ASD fecal LPS samples. Results reported in the table derive by combining of data from compositional analysis, through acetylated methyl glycosides derivatization, and absolute configuration, through acetylated octyl glycosides derivatization. Rha: rhamnose; Fuc: fucose; Ara: arabinose; Xyl: xylose; GalA: galacturonic acid; Man: mannose; Gal: galactose; Glc: glucose; GalN: galactosamine; GlcN: glucosamine; Hep: heptose; Kdo: 3-deoxy-D-*manno*-oct-2-ulosonic acid

	**Monosaccharide composition**
**NASD1-9/12**	L-Rha; L-Fuc; D-Man; D-Gal; D-Glc; D-GalN; D-GlcN; Kdo
**NASD10-11**	L-Rha; D-Man; D-Gal; D-Glc; D-GlcN; Kdo
**ASD1**	L-Rha; L-Fuc; D-GalA; D-Man; D-Gal; D-Glc; D-GalN; D-GlcN; L,D-Hep; Kdo
**ASD2**	L-Rha; L-Fuc; D-GalA; D-Man; D-Gal; D-Glc; D-GalN; D-GlcN; Kdo
**ASD3**	L-Rha; L-Fuc; L-Ara; D-Man; D-Gal; D-Glc; D-GalN; D-GlcN; L,D-Hep; Kdo
**ASD4**	L-Rha; D-Man; D-Gal; D-Glc; D-GlcN; L,D-Hep; Kdo
**ASD5**	L-Rha; L-Ara; D-Xyl; D-GalA; D-Man; D-Gal; D-Glc; D-GalN; D-GlcN; Kdo
**ASD6**	L-Rha; L-Fuc; D-Man; D-Gal; D-Glc; D-GlcN; Kdo
**ASD7**	L-Rha; D-GalA; D-Man; D-Gal; D-Glc; D-GalN; D-GlcN; Kdo
**ASD8**	L-Rha; L-Fuc; D-GalA; D-Man; D-Gal; D-Glc; D-GalN; D-GlcN; L,D-Hep; Kdo
**ASD9**	L-Rha; L-Fuc; D-Man; D-Gal; D-Glc; D-GalN; D-GlcN; L,D-Hep; Kdo
**ASD10**	L-Rha; D-Man; D-Gal; D-Glc; D-GlcN; L,D-Hep; Kdo
**ASD11**	L-Rha; L-Ara; D-GalA; D-Man; D-Gal; D-Glc; D-GalN; D-GlcN; L,D-Hep; Kdo
**ASD12**	L-Rha; L-Fuc; D-Man; D-Gal; D-Glc; D-GlcN; Kdo

Table S2. Bacterial proteins identified in each sample with their respective biological function.

Large ribosomal subunit protein bL19	*rplS*	Protein synthesis
Large ribosomal subunit protein uL11	*rplK*	Protein synthesis
Large ribosomal subunit protein uL2	*rplB*	Protein synthesis
Large ribosomal subunit protein uL22	*rplV*	Protein synthesis
Orotidine 5'-phosphate decarboxylase	*pyrF*	Protein synthesis
Small ribosomal subunit protein bS6	*rpsF*	Protein synthesis
3-hydroxyacyl-[acyl-carrier-protein] dehydratase FabZ	*fabZ*	SCFAs metabolism
3-hydroxybutyryl-CoA dehydrogenase	*hbd*	SCFAs metabolism
3-keto-disaccharide hydrolase		SCFAs metabolism
4-deoxy-L-threo-5-hexosulose-uronate ketol-isomerase 1	*kduI1*	SCFAs metabolism
4-deoxy-L-threo-5-hexosulose-uronate ketol-isomerase 2	*kduI2*	SCFAs metabolism
Acetate kinase	*ackA*	SCFAs metabolism
Acetyl-CoA acetyltransferase	*thlA*	SCFAs metabolism
Acyl-CoA dehydrogenase	*acdA*	SCFAs metabolism
Malate dehydrogenase	*mdh*	SCFAs metabolism
Xylose isomerase	*xylA*	SCFAs metabolism
Alkaline phosphatase L	*lapA*	Stress/redox response
Alkyl hydroperoxide reductase C	*ahpC*	Stress/redox response
Bacterial non-heme ferritin	*ftnA*	Stress/redox response
Catalase HPII	*katE*	Stress/redox response
Chaperone protein DnaK	*dnaK*	Stress/redox response
DNA protection during starvation protein	*dps*	Stress/redox response
DNA-binding protein HU-alpha	*hupA*	Stress/redox response
DNA-binding protein HU-beta	*hupB*	Stress/redox response
Superoxide dismutase [Fe]	*sodB*	Stress/redox response

Table S3. Differential protein expression.

Up-regulated proteins					
**Swiss Code**	**Protein name**	**Gene Name**	**Biological function**	**Linear FC**	**Log_2 FC**
0.48B9DK67	Malate dehydrogenase	*mdh*	lactate metabolic process; tricarboxylic acid cycle	1.4	0.44
Q8A767	Probable transaldolase	*tal*	carbohydrate metabolic process; pentose-phosphate shunt	1.3	0.41
Q18CH0	Small ribosomal subunit protein uS8	*rpsH*	translation	1.3	0.40
Q8G485	Elongation factor Ts	*tsf*	protein biosynthesis	1.3	0.40
P13794	Outer membrane porin F	*oprF*	regulation of cell shape	1.3	0.39
Q06879	Pyruvate-flavodoxin oxidoreductase	*nifJ*	electron transport chain; response to oxidative stress	1.3	0.39
B7GS74	Large ribosomal subunit protein bL25	*rplY*	translation	1.3	0.39
O50306	Elongation factor Tu	*tuf*	protein biosynthesis	1.3	0.39
C4Z8W9	2-C-methyl-D-erythritol-2,4-cyclodiphosphate synthase	*ispF*	biosynthetic process	1.3	0.38
C4ZBU5	Small ribosomal subunit protein uS4	*rpsD*	translation	1.3	0.37
P31206	Sialidase	*nanH*	catabolic process	1.3	0.35
Q64XV7	Phosphoribosylaminoimidazole-succinocarboxamide synthase	*purC*	biosynthetic process	1.3	0.35
Q7MZ27	Probable cytosol aminopeptidase	*pepA*	proteolysis	1.3	0.35
Q8A155	Bifunctional purine biosynthesis protein PurH	*purH*	biosynthetic process	1.3	0.34
Q6AP73	Elongation factor Tu 2	*tuf2*	protein biosynthesis	1.3	0.34

P0A4J4	Superoxide dismutase [Mn/Fe]	*sodA*	oxidoreductase (mol)	1.3	0.33
Q18CJ5	Small ribosomal subunit protein uS9	*rpsI*	translation	1.3	0.32
B0RZS8	Large ribosomal subunit protein uL15	*rplO*	translation	1.3	0.32
A6TWH6	Small ribosomal subunit protein uS3	*rpsC*	translation	1.2	0.32
A6KYY1	Glycosyl hydrolase family 109 protein 3		glycosidase (mol)	1.2	0.31
Q8A0B5	4-deoxy-L-threo-5-hexosulose-uronate ketol-isomerase 2	*kduI2*	catabolic process	1.2	0.31
Q87BU0	Phosphoserine aminotransferase	*serC*	biosynthetic process	1.2	0.30
A6LPU6	Large ribosomal subunit protein uL13	*rplM*	translation	1.2	0.30
B8DW24	Large ribosomal subunit protein uL24	*rplX*	translation	1.2	0.29
O05239	Probable NADH-dependent butanol dehydrogenase 1	*yugJ*	1-butanol biosynthetic process	1.2	0.29
A6LFI7	UPF0145 protein BDI_2732			1.2	0.29
Q65I26	Nucleoside diphosphate kinase	*ndk*	biosynthetic process	1.2	0.28
O34525	Putative signal peptide peptidase SppA	*sppA*	proteolysis	1.2	0.28
Q8A1G0	Neopullulanase SusA	*susA*	catabolic process	1.2	0.28
Q6NID8	Bifunctional purine biosynthesis protein PurH	*purH*	biosynthetic process	1.2	0.28
Q5YPG2	Small ribosomal subunit protein uS7	*rpsG*	translation	1.2	0.28
A8ZRR1	Flotillin-like protein FloA	*floA*	membrane fluidity	1.2	0.27
Q0SQC8	Elongation factor Tu	*tuf1*	protein biosynthesis	1.2	0.27
A1A040	Co-chaperonin GroES	*groES*	protein refolding	1.2	0.27
Q18CG7	Large ribosomal subunit protein uL14	*rplN*	translation	1.2	0.26

G3KIM3	Lactoyl-CoA dehydratase subunit beta (Fragment)	*lcdB*	lactoyl-CoA dehydratase activity (mol)	1.2	0.26
Q31IX0	Large ribosomal subunit protein uL5	*rplE*	translation	1.2	0.25
B1IRU4	Autoinducer 2-binding protein LsrB	*lsrB*	transmembrane transport	1.2	0.25
Q8A9E3	Aspartate--tRNA ligase	*aspS*	aspartyl-tRNA aminoacylation	1.2	0.25
P47545	Putative ABC transporter ATP-binding protein MG303		transport	1.2	0.25
B7GJ73	Small ribosomal subunit protein uS3	*rpsC*	translation	1.2	0.24
Q8R7W0	Small ribosomal subunit protein uS3	*rpsC*	translation	1.2	0.24
Q8A753	Phosphoglycerate kinase	*pgk*	gluconeogenesis ; glycolytic process	1.2	0.24
Q71WB9	Elongation factor Tu	*tuf*	protein biosynthesis	1.2	0.24
A7Z207	Chaperonin GroEL	*groEL*	protein refolding	1.2	0.24
Q8XHS8	Large ribosomal subunit protein uL22	*rplV*	translation	1.2	0.24
A6L048	L-fucose isomerase	*fucI*	catabolic process	1.2	0.24
Q5WLM8	Small ribosomal subunit protein uS9	*rpsI*	translation	1.2	0.24
B3PME9	Elongation factor G	*fusA*	ribosome disassembly	1.2	0.23
Q65E69	4-deoxy-L-threo-5-hexosulose-uronate ketol-isomerase	*kduI*	catabolic process	1.2	0.23
P0DUM6	Bacterial microcompartment shell protein PduA	*pduA*	catabolic process	1.2	0.23
O32167	Methionine-binding lipoprotein MetQ	*metQ*	amino acid transport	1.2	0.23
A8DEZ8	Proline racemase		isomerase (mol)	1.2	0.23
P19994	Methionine aminopeptidase 1	*map*	proteolysis	1.2	0.22
C4ZBS9	Large ribosomal subunit protein uL14	*rplN*	translation	1.2	0.22

B7GNG8	Large ribosomal subunit protein bL12	*rplL*	translation	1.2	0.22
Q9I165	Periplasmic trehalase	*treA*	cellular catabolic process; cellular hyperosmotic response	1.2	0.22
P14949	Thioredoxin	*trxA*	cell redox homeostasis	1.2	0.22
P64028	Elongation factor Tu	*tuf*	protein biosynthesis	1.2	0.22
C5B747	Large ribosomal subunit protein uL13	*rplM*	translation	1.2	0.21
A9ACU4	Glycine cleavage system H protein	*gcvH*	glycine decarboxylation via glycine cleavage system	1.2	0.21
A8F916	Putative septation protein SpoVG	*spoVG*	division septum assembly	1.2	0.21
A8FG47	Malate dehydrogenase	*mdh*	lactate metabolic process; tricarboxylic acid cycle	1.2	0.21
P45040	Cysteine synthase	*cysK*	biosynthetic process	1.2	0.21
P0C863	Glycosyl hydrolase family 109 protein 1	*BF0931*	glycosidase (mol)	1.2	0.21
Q1GZ16	Formate--tetrahydrofolate ligase	*fhs*	tetrahydrofolate interconversion	1.2	0.21
B9DYC1	Large ribosomal subunit protein uL5	*rplE*	translation	1.2	0.21
Q38UH2	L-arabinose isomerase	*araA*	catabolic process	1.2	0.21
A4WFB5	Small ribosomal subunit protein uS14	*rpsN*	translation	1.2	0.21
**Down-regulated proteins**					
**Swiss Code**	**Protein Name**	**Gene Name**	**Biological function**	**FC**	**Log_2FC**
P45364	3-hydroxybutyryl-CoA dehydrogenase	*hbd*	butyrate metabolic process; fatty	0.3	-1.96

			acid beta-oxidation		
P0A6A5	Acetate kinase	*ackA*	acetate metabolic process	0.3	-1.60
P0A9G6	Isocitrate lyase	*aceA*	tricarboxylic acid cycle	0.4	-1.32
P45867	Acyl-CoA dehydrogenase	*acdA*	fatty acid beta-oxidation	0.4	-1.25
A8FI72	3-hydroxyacyl-[acyl-carrier-protein] dehydratase FabZ	*fabZ*	fatty acid biosynthetic process	0.5	-1.03
Q8A0W0	Malate dehydrogenase	*mdh*	tricarboxylic acid cycle	0.5	-0.96
A6L903	Malate dehydrogenase	*mdh*	lactate metabolic process; tricarboxylic acid cycle	0.6	-0.69
Q9AEM9	Xylulose-5-phosphate/fructose-6-phosphate phosphoketolase	*xfp*	carbohydrate metabolic process	0.7	-0.56
P58072	Glyceraldehyde-3-phosphate dehydrogenase C	*gapC*	glycolytic process	0.7	-0.46
B1IGF8	Small ribosomal subunit protein uS7	*rpsG*	translation	0.8	-0.40
Q838I4	Superoxide dismutase [Fe]	*sodA*	oxidoreductase (mol)	0.8	-0.39
P0A392	Leucine dehydrogenase	*ldh*	catabolic process	0.8	-0.37
P24750	Glyceraldehyde-3-phosphate dehydrogenase (Fragment)	*gap*	glycolytic process	0.8	-0.37
Q73P70	Ornithine carbamoyltransferase, catabolic	*arcB*	biosynthetic process	0.8	-0.37
P58228	Glutamate decarboxylase alpha	*gadA*	glutamate catabolic process	0.8	-0.36
Q6HAG9	Adenylosuccinate synthetase	*purA*	metabolic process	0.8	-0.34
P0A9Q7	Bifunctional aldehyde-alcohol dehydrogenase AdhE	*adhE*	response to oxidative stress	0.8	-0.33

B1HQI0	Small ribosomal subunit protein bS16	*rpsP*	translation	0.8	-0.33
C5D4J5	1-pyrroline-5-carboxylate dehydrogenase	*rocA*	glutamate biosynthetic process	0.8	-0.33
P0AEB7	RutC family protein YoaB	*yoaB*	biofilm formation	0.8	-0.33
O32755	Glyceraldehyde-3-phosphate dehydrogenase	*gap*	glycolytic process	0.8	-0.33
P61951	Flavodoxin 1	*fldA*	electron transport	0.8	-0.33
A0R1Y7	Probable acetyl-CoA acetyltransferase		acyltransferase (mol)	0.8	-0.32
P80505	Glyceraldehyde-3-phosphate dehydrogenase 2	*gap2*	glycolytic process	0.8	-0.32
Q8G3Q1	Xylose isomerase	*xylA*	metabolic process	0.8	-0.32
B1LHV9	Common pilus major fimbrillin subunit EcpA	*ecpA*	biofilm development	0.8	-0.31
P99068	Nucleoside diphosphate kinase	*ndk*	biosynthetic process	0.8	-0.31
P0A957	KHG/KDPG aldolase	*eda*	carbohydrate metabolism	0.8	-0.31
Q9REM8	Adhesin P1	*gapA*	adhesion of symbiont to microvasculature	0.8	-0.31
Q9ZFV8	NADP-dependent malic enzyme	*maeB*	malate metabolic process	0.8	-0.30
Q7VQX0	Histidinol dehydrogenase	*hisD*	biosynthetic process	0.8	-0.30
B1GZ68	Uronate isomerase	*uxaC*	catabolic process	0.8	-0.30
Q38UT8	Large ribosomal subunit protein bL17	*rplQ*	translation	0.8	-0.29
Q65GM5	Large ribosomal subunit protein bL27	*rpmA*	translation	0.8	-0.29
A9MHA7	Glucose-6-phosphate isomerase	*pgi*	gluconeogenesis	0.8	-0.29
P0AB73	Fructose-bisphosphate aldolase class 2	*fbaA*	gluconeogenesis	0.8	-0.29

A6LSS9	6,7-dimethyl-8-ribityllumazine synthase	*ribH*	biosynthetic process	0.8	-0.29
B0CDA0	Histidine--tRNA ligase	*hisS*	histidyl-tRNA aminoacylation	0.8	-0.28
Q5KUI2	Serine hydroxymethyltransferase	*glyA*	glycine biosynthetic process	0.8	-0.28
Q65HL2	Bacilliredoxin BLi02578/BL01507		cell redox homeostasis; response to oxidative stress	0.8	-0.28
P0AE08	Alkyl hydroperoxide reductase C	*ahpC*	cell redox homeostasis; response to oxidative stress	0.8	-0.27
A0ALD9	Enolase	*eno*	glycolytic proces	0.8	-0.27
B1HNL5	Large ribosomal subunit protein uL1	*rplA*	translation	0.8	-0.27
B4EYV0	Large ribosomal subunit protein bL12	*rplL*	translation	0.8	-0.27
Q8FJM0	DNA protection during starvation protein	*dps*	chromosome condensation; intracellular iron ion homeostasis	0.8	-0.27
P0AFK1	Metalloprotease PmbA	*pmbA*	proteolysis	0.8	-0.27
Q64N36	Glycine cleavage system H protein	*gcvH*	glycine decarboxylation via glycine cleavage system	0.8	-0.26
Q6GEV0	Fructose-bisphosphate aldolase	*fba*	glycolytic process	0.8	-0.26
Q7MYB3	Triosephosphate isomerase	*tpiA*	gluconeogenesis	0.8	-0.26
Q83IN9	Glucose-6-phosphate isomerase	*pgi*	gluconeogenesis	0.8	-0.26
P0A913	Peptidoglycan-associated lipoprotein	*pal*	cell division	0.8	-0.26
B5YTP2	Small ribosomal subunit protein uS10	*rpsJ*	translation	0.8	-0.26
Q5PK21	Phosphopentomutase	*deoB*	metabolic process	0.8	-0.26

P0AEX9	Maltose/maltodextrin-binding periplasmic protein	*malE*	carbohydrate transport cell chemotaxis; DNA damage response	0.8	-0.26
A1A399	Beta-galactosidase BgaB	*bgaB*	carbohydrate metabolic process	0.8	-0.25
Q05203	Flagellin	*hag*	structural molecule activity (mol)	0.8	-0.25
P05806	Bacillolysin	*npr*	proteolysis	0.8	-0.25
A8FAS1	Aspartyl/glutamyl-tRNA(Asn/Gln) amidotransferase subunit B	*gatB*	translation	0.8	-0.25
Q1H4N9	Elongation factor Tu 2	*tuf2*	protein biosynthesis	0.8	-0.25
Q83MH5	Chaperone protein DnaK	*dnaK*	protein folding	0.8	-0.25
Q7MX30	Large ribosomal subunit protein uL1	*rplA*	translation	0.8	-0.25
A7ZUD3	Triosephosphate isomerase	*tpiA*	gluconeogenesis	0.8	-0.25
Q46939	Probable acetyl-CoA acetyltransferase	*yqeF*	1-butanol biosynthetic process	0.8	-0.24
Q8CVC9	Phosphate-binding protein PstS 2	*pstS2*	phosphate ion transport	0.8	-0.24
P08066	Succinate dehydrogenase iron-sulfur subunit	*sdhB*	aerobic respiration; respiratory electron transport chain; tricarboxylic acid cycle	0.8	-0.24
P09394	Glycerophosphodiester phosphodiesterase, periplasmic	*glpQ*	lipid metabolic process	0.8	-0.24
P36772	Lon protease	*lon*	cellular response to heat; protein quality control for misfolded or incompletely synthesized proteins	0.8	-0.24

Q8FD58	N-acetylneuraminate lyase 1	*nanA1*	carbohydrate metabolic process	0.8	-0.24
B7GNH2	Polyribonucleotide nucleotidyltransferase	*pnp*	mRNA catabolic process; RNA processing	0.8	-0.24
P69783	PTS system glucose-specific EIIA component	*crr*	carbohydrate metabolic process	0.8	-0.24
P24751	Glyceraldehyde-3-phosphate dehydrogenase (Fragment)	*gap*	glycolytic process	0.8	-0.24
Q06282	Glycerophosphodiester phosphodiesterase	*glpQ*	lipid metabolic process	0.8	-0.23
